# Evaluation of Fecal Inflammatory Biomarkers to Identify Bacterial Diarrhea Episodes: Systematic Review and Protocol for the Enterics for Global Health *Shigella* Surveillance Study

**DOI:** 10.1093/ofid/ofad652

**Published:** 2024-03-25

**Authors:** Courtney Babb, Henry Badji, Md Taufiqur Rahman Bhuiyan, Jennifer Cornick, Sonia Qureshi, Catherine Sonye, Wagner V Shapiama Lopez, Mehreen Adnan, Hannah E Atlas, Kehkashan Begum, Stephanie A Brennhofer, Bubacarr E Ceesay, Abdoulie K Ceesay, Nigel A Cunliffe, Paul F Garcia Bardales, Shahinur Haque, Bri’Anna Horne, M Jahangir Hossain, Junaid Iqbal, Md Taufiqul Islam, Sadia Islam, Farhana Khanam, Karen L Kotloff, Thandizo Malemia, Katia Manzanares Villanueva, Gertrude Malola Million, Vitumbiko Munthali, John Benjamin Ochieng, Billy Ogwel, Maribel Paredes Olortegui, Richard Omore, Patricia B Pavlinac, James A Platts-Mills, Khandra T Sears, Ousman Secka, Sharon M Tennant, Pablo Peñataro Yori, Mohammad Tahir Yousafzai, Khuzwayo C Jere, Margaret N Kosek, Stephen Munga, Usman N Ikumapayi, Firdausi Qadri, Farah Naz Qamar, Elizabeth T Rogawski McQuade

**Affiliations:** Department of Epidemiology, Emory University, Atlanta, Georgia, USA; Medical Research Council Unit The Gambia, London School of Hygiene and Tropical Medicine, Fajara, The Gambia; Infectious Diseases Division, International Centre for Diarrhoeal Disease Research, Bangladesh, Dhaka, Bangladesh; Institute of Infection, Veterinary and Ecological Sciences, Department of Clinical Infection, Microbiology and Immunology, University of Liverpool, Liverpool, United Kingdom; Malawi Liverpool Wellcome Programme, Blantyre, Malawi; Department of Pediatrics and Child Health, The Aga Khan University, Karachi, Pakistan; Center for Global Health Research, Kenya Medical Research Institute, Kisumu, Kenya; Asociación Benéfica PRISMA, Iquitos, Peru; Department of Pediatrics and Child Health, The Aga Khan University, Karachi, Pakistan; Department of Global Health, University of Washington, Seattle, Washington, USA; Department of Pediatrics and Child Health, The Aga Khan University, Karachi, Pakistan; Division of Infectious Diseases and International Health, University of Virginia, Charlottesville, Virginia, USA; Medical Research Council Unit The Gambia, London School of Hygiene and Tropical Medicine, Fajara, The Gambia; Medical Research Council Unit The Gambia, London School of Hygiene and Tropical Medicine, Fajara, The Gambia; Institute of Infection, Veterinary and Ecological Sciences, Department of Clinical Infection, Microbiology and Immunology, University of Liverpool, Liverpool, United Kingdom; Asociación Benéfica PRISMA, Iquitos, Peru; Infectious Diseases Division, International Centre for Diarrhoeal Disease Research, Bangladesh, Dhaka, Bangladesh; Center for Vaccine Development and Global Health, University of Maryland School of Medicine, Baltimore, Maryland, USA; Department of Medicine, University of Maryland School of Medicine, Baltimore, Maryland, USA; Medical Research Council Unit The Gambia, London School of Hygiene and Tropical Medicine, Fajara, The Gambia; Department of Pediatrics and Child Health, The Aga Khan University, Karachi, Pakistan; Infectious Diseases Division, International Centre for Diarrhoeal Disease Research, Bangladesh, Dhaka, Bangladesh; Infectious Diseases Division, International Centre for Diarrhoeal Disease Research, Bangladesh, Dhaka, Bangladesh; Infectious Diseases Division, International Centre for Diarrhoeal Disease Research, Bangladesh, Dhaka, Bangladesh; Center for Vaccine Development and Global Health, University of Maryland School of Medicine, Baltimore, Maryland, USA; Department of Medicine, University of Maryland School of Medicine, Baltimore, Maryland, USA; Malawi Liverpool Wellcome Programme, Blantyre, Malawi; Asociación Benéfica PRISMA, Iquitos, Peru; Malawi Liverpool Wellcome Programme, Blantyre, Malawi; Malawi Liverpool Wellcome Programme, Blantyre, Malawi; Center for Global Health Research, Kenya Medical Research Institute, Kisumu, Kenya; Center for Global Health Research, Kenya Medical Research Institute, Kisumu, Kenya; Asociación Benéfica PRISMA, Iquitos, Peru; Center for Global Health Research, Kenya Medical Research Institute, Kisumu, Kenya; Department of Global Health, University of Washington, Seattle, Washington, USA; Division of Infectious Diseases and International Health, University of Virginia, Charlottesville, Virginia, USA; Center for Vaccine Development and Global Health, University of Maryland School of Medicine, Baltimore, Maryland, USA; Department of Medicine, University of Maryland School of Medicine, Baltimore, Maryland, USA; Medical Research Council Unit The Gambia, London School of Hygiene and Tropical Medicine, Fajara, The Gambia; Center for Vaccine Development and Global Health, University of Maryland School of Medicine, Baltimore, Maryland, USA; Department of Medicine, University of Maryland School of Medicine, Baltimore, Maryland, USA; Division of Infectious Diseases and International Health, University of Virginia, Charlottesville, Virginia, USA; Department of Pediatrics and Child Health, The Aga Khan University, Karachi, Pakistan; Institute of Infection, Veterinary and Ecological Sciences, Department of Clinical Infection, Microbiology and Immunology, University of Liverpool, Liverpool, United Kingdom; Malawi Liverpool Wellcome Programme, Blantyre, Malawi; Department of Medical Laboratory Sciences, Kamuzu University of Health Sciences, School of Life Sciences and Health Professions, Blantyre, Malawi; Division of Infectious Diseases and International Health, University of Virginia, Charlottesville, Virginia, USA; Center for Global Health Research, Kenya Medical Research Institute, Kisumu, Kenya; Medical Research Council Unit The Gambia, London School of Hygiene and Tropical Medicine, Fajara, The Gambia; Infectious Diseases Division, International Centre for Diarrhoeal Disease Research, Bangladesh, Dhaka, Bangladesh; Department of Pediatrics and Child Health, The Aga Khan University, Karachi, Pakistan; Department of Epidemiology, Emory University, Atlanta, Georgia, USA

**Keywords:** diagnostic, diarrhea, inflammatory biomarker, *Shigella*, systematic review

## Abstract

**Background:**

The measurement of fecal inflammatory biomarkers among individuals presenting to care with diarrhea could improve the identification of bacterial diarrheal episodes that would benefit from antibiotic therapy. We reviewed prior literature in this area and describe our proposed methods to evaluate 4 biomarkers in the Enterics for Global Health (EFGH) *Shigella* surveillance study.

**Methods:**

We systematically reviewed studies since 1970 from PubMed and Embase that assessed the diagnostic characteristics of inflammatory biomarkers to identify bacterial diarrhea episodes. We extracted sensitivity and specificity and summarized the evidence by biomarker and diarrhea etiology. In EFGH, we propose using commercial enzyme-linked immunosorbent assays to test for myeloperoxidase, calprotectin, lipocalin-2, and hemoglobin in stored whole stool samples collected within 24 hours of enrollment from participants in the Bangladesh, Kenya, Malawi, Pakistan, Peru, and The Gambia sites. We will develop clinical prediction scores that incorporate the inflammatory biomarkers and evaluate their ability to identify *Shigella* and other bacterial etiologies of diarrhea as determined by quantitative polymerase chain reaction (qPCR).

**Results:**

Forty-nine studies that assessed fecal leukocytes (n = 39), red blood cells (n = 26), lactoferrin (n = 13), calprotectin (n = 8), and myeloperoxidase (n = 1) were included in the systematic review. Sensitivities were high for identifying *Shigella*, moderate for identifying any bacteria, and comparable across biomarkers. Specificities varied depending on the outcomes assessed. Prior studies were generally small, identified red and white blood cells by microscopy, and used insensitive gold standard diagnostics, such as conventional bacteriological culture for pathogen detection.

**Conclusions:**

Our evaluation of inflammatory biomarkers to distinguish diarrhea etiologies as determined by qPCR will provide an important addition to the prior literature, which was likely biased by the limited sensitivity of the gold standard diagnostics used. We will determine whether point-of-care biomarker tests could be a viable strategy to inform treatment decision making and increase appropriate targeting of antibiotic treatment to bacterial diarrhea episodes.

## Teaser

Fecal inflammatory biomarkers may accurately identify bacterial diarrheal episodes, which could inform antibiotic treatment decisions. Prior research was limited by using insensitive diagnostics. We will evaluate 4 inflammatory biomarkers in stool from children with diarrhea in the EFGH study.

Bacterial pathogens, such as *Shigella* and diarrheagenic *Escherichia coli*, are leading causes of diarrhea among children <5 years of age in low-resource settings. Appropriate antibiotic treatment of bacterial diarrhea episodes can limit morbidity and mortality [1, 2]. In a large, multicountry trial of azithromycin for children with watery diarrhea and dehydration, severe stunting, or moderate wasting, the benefit of azithromycin was observed primarily in children with a bacterial cause of diarrhea, namely *Campylobacter*, typical enteropathogenic *E coli* (EPEC), heat-stable enterotoxigenic *E coli* (ST-ETEC), *Salmonella*, *Shigella*, and *Vibrio cholerae* [3]. Furthermore, antibiotic treatment of *Shigella*-attributed moderate-to-severe diarrhea (MSD) was associated with improved short-term linear growth in the Global Enteric Multicenter Study (GEMS) [4], and antibiotic treatment of MSD was associated with lower risk of persistent diarrhea in the Vaccine Impact on Diarrhea in Africa (VIDA) study [5].

Targeting antibiotics to children with bacterial diarrhea is needed to limit antibiotic overuse and development of antimicrobial resistance. Current treatment guidelines take a syndromic approach, recommending antibiotic treatment for dysentery or presumed cholera [6], which comprise a small proportion of all bacterial diarrhea episodes. In the Etiology, Risk Factors, and Interactions of Enteric Infections and Malnutrition and the Consequences for Child Health and Development (MAL-ED) and VIDA studies, caregivers reported blood in stool for only 14.5% [7] and 43.8% [8] of shigellosis episodes, respectively. Of all bacterial diarrhea episodes in MAL-ED, caregivers reported blood in 10.4%. This suggests that most cases of shigellosis and other bacterial diarrhea episodes are missed according to current guidelines. Furthermore, younger children, who are most likely to die or be hospitalized from diarrhea and could significantly benefit from treatment, are less likely to present with dysentery [8–10]. Watery bacterial episodes are difficult to distinguish clinically, and prediction scores for specific etiologies that have been developed tend to be driven more heavily by epidemiologic characteristics (including age and season) than the presence of symptoms. For example, a clinical prediction score for *Shigella* developed in MAL-ED classified that nearly all episodes among children >18 months should be treated, and identified only a few episodes from 6–18 months that should be treated depending primarily on the presence of blood in addition to other symptoms [7]. While it is an important improvement over using the presence of blood alone to identify shigellosis, this score still only identified half of *Shigella*-attributed episodes [7].

In the absence of readily available point-of-care (POC) diagnostics, measurement of inflammatory biomarkers (ie, those indicative of leukocytes and/or erythrocytes in stool) could substantially improve clinical prediction scores to identify the subset of watery diarrhea episodes that would benefit from antibiotic therapy. *Shigella* secretes virulence factors that have enterotoxic activity and allow *Shigella* to invade the colonic epithelium, inducing an inflammatory response [11]. *Campylobacter, Salmonella*, enteroaggregative *E coli*, EPEC, and enteroinvasive *E coli* are also inflammatory and can cause invasive disease [12]. Several studies beginning in the 1970s found that the presence of red blood cells (RBCs) and white blood cells (WBCs) on stool microscopy was more common in shigellosis and other bacterial diarrhea episodes compared to viral episodes [13–18], and immunoassays for biomarkers of leukocytes such as lactoferrin [19–22] and calprotectin [23–27] have also been assessed to distinguish diarrhea etiology. If these markers prove to be sufficiently predictive of watery bacterial diarrhea episodes, inflammatory biomarker stool tests could be adapted into lateral flow assays, which would be readily deployable at the POC to inform antibiotic treatment, with limited demand for staff training or laboratory infrastructure.

The Enterics for Global Health (EFGH) *Shigella* surveillance study offers an ideal platform to further investigate novel strategies to identify bacterial diarrhea given its rich dataset and sample archive among a geographically diverse sample of children with diarrhea. In this article, we systematically review the literature of studies that assessed the sensitivity and specificity of fecal inflammatory biomarkers to identify bacterial diarrhea and describe our proposed methods for characterizing the performance of inflammatory biomarker tests to identify watery shigellosis and other bacterial diarrhea episodes in EFGH.

## METHODS

We searched PubMed and Embase databases for studies published after 1 January 1970, using a combination of search terms (Supplementary Appendix) to capture bacterial diarrhea, fecal inflammatory biomarkers, and diagnostic studies. Included studies were published in English and conducted in individuals of all ages with bacterial diarrhea in any setting. Studies of natural history, blood biomarkers, diarrheal illnesses related to chronic diseases, asymptomatic infections, and animals were excluded. Review articles, case reports, and studies without sufficient data for extraction were also excluded.

Study selection was conducted using Covidence software [28]. Screening of titles and abstracts and the full text review was performed independently by 2 reviewers (C. B., S. Q., H. B., W. V. S. L., or E. T. R. M.). Disagreements were resolved by a third reviewer. Enrollment dates, study location, number of participants with diarrhea and specific pathogens, diagnostic gold standard used, biomarkers assessed, and the associated sensitivities and specificities for the identification of *Shigella* and a combined bacterial diarrhea outcome if available (eg, all bacteria, invasive bacteria, or a group of specific pathogens; Supplementary Table 1), were extracted from included studies by 1 of the above authors and checked by a second author (C. B. or E. T. R. M.). This review is registered with PROSPERO (CRD42023409479).

If a combined bacterial diarrhea outcome was not reported, we summed counts of individual bacterial outcomes and calculated sensitivity for the group of bacteria. When specificity was not directly provided, we calculated specificity as 1-sensitivity for the detection of negative outcomes such as no pathogen detected, viruses or parasites detected, or noninflammatory or noninvasive bacteria detected (Supplementary Table 1). We categorized studies by setting and age group. The quality of studies was assessed using Quality Assessment of Diagnostic Accuracy Studies (QUADAS) criteria [29] (Supplementary Table 2).

## RESULTS

After screening 3875 titles and abstracts, and reviewing 93 full texts, 49 studies met inclusion criteria (Supplementary Figure 1). The enrollment period of included studies ranged from 1975 to 2020 (Supplementary Table 1). Thirty-four (69%) studies included children, 12 studies (25%) included adults only, and age was unknown for 3 (6%) studies. Twenty-four (49%) studies were conducted in low- and middle-income countries (LMICs) compared to 25 (51%) in high-income countries. Four studies were of travelers or the military, and 1 study included experimentally infected healthy volunteers. Of the 49 studies included, 39 (80%) assessed fecal leukocytes, 26 (53%) assessed RBCs/occult blood, 13 (27%) assessed lactoferrin, 8 (16%) assessed calprotectin, and 1 (2%) assessed myeloperoxidase (MPO). *Shigella* was assessed in 21 (43%) studies, and other bacterial outcomes were assessed in 40 (82%) studies. Eighteen (36%) studies included children in LMICs, of which 17 (35%) examined leukocytes, 10 (20%) examined RBCs/occult blood, and 5 (10%) examined other biomarkers. Most studies (n = 43 [88%]) used traditional bacterial culture alone as the gold standard diagnostic to identify bacterial causes of diarrhea. Three studies (6%) used culture and polymerase chain reaction (PCR), and 3 studies (6%) used PCR alone.

The median sensitivity of fecal leukocytes for identifying *Shigella* was 78% (interquartile range [IQR], 68%–94%) and 45% (IQR, 33%–69%) for identifying combined bacterial outcomes (Table 1). Specificity for *Shigella* was assessed in only 3 studies and ranged from 61% to 74%. Specificity for combined bacterial outcomes varied depending on the nonbacterial outcomes considered, with a median specificity of 85% (IQR, 75%–90%). For RBCs/occult blood, median sensitivity for *Shigella* was 70% (IQR, 51%–84%) and for combined bacterial outcomes was 48% (IQR, 37%–74%; Table 2). Specificity for *Shigella* was estimated in 1 study at 61% [30], and median specificity for combined bacterial outcomes was 69% (IQR, 63%–85%). Sensitivity of lactoferrin for *Shigella* was estimated in 4 studies and ranged from 61% to 100% (Table 3). Median sensitivity and specificity of lactoferrin for combined bacterial outcomes was 88% (IQR, 74%–94%) and 51% (IQR, 26%–69%), respectively. For calprotectin, sensitivity for *Shigella* was 78% in 1 study. Median sensitivity and specificity of calprotectin for combined bacterial outcomes was 86% (IQR, 76%–93%) and 69% (IQR, 41%–87%), respectively. The 1 study that assessed MPO estimated sensitivity of 78% and specificity of 57% for combined bacterial outcomes [31]. In the 2 studies that assessed both lactoferrin and calprotectin, sensitivity for combined bacterial outcomes was higher for calprotectin [26, 31]. Specificity was not consistently higher or lower between the 2 biomarkers (Table 3) [26, 31].

**Table 1.** Sensitivity and Specificity of Fecal Leukocytes to Identify *Shigella* and Other Bacterial Causes of Diarrhea in Systematically Reviewed Studies by Setting and Age of Included Individuals (n = 39 Studies)

Setting and Study	Study Location	WBC Cutoff (per HPF^a^)	No. With Diarrhea	No. With *Shigella*	Sensitivity for *Shigella*, %	Specificity for *Shigella*, %	No. With Combined Bacterial Outcome	Sensitivity for Combined Bacterial Outcome, %	Specificity for Combined Bacterial Outcome^b^, %
LMIC children (n = 17)									
Bardhan 2000 [43]	Bangladesh	>20	1008	205	63	…	…	…	86–96
Beltinger 1997 [44]	Bangladesh	>20	304	38	76	…	54	67	87
Bodhidatta 2002 [45]	Thailand	>10	623	56	83	…	…	…	…
Chang 2017 [46]	China	≥5	680	1	100	…	215	23	94
Huicho 1993 [16]	Peru	>5	446	10	70	…	58	36	82–88
Huicho 1997 [21]	Peru	NS	125	6	…	…	29	69	60
Ismail 1994 [30]	Indonesia	>5	619	44	68	61	60	52	60
Jindal 1991 [47]	India	>10	400	6	67	…	115	28	88
Khan 2006 [17]	Bangladesh	>20	843	454	71	62	…	…	…
Korzeniowski 1979 [14]	USA, Brazil	>10/LPF	101	29	95	…	…	…	85
McNeely 1996 [48]	Mexico	>5	1040	143	…	…	173	42	76
Mercado 2011 [18]	Peru	>10	935	…	…	…	257	14	90
Mshana 2010 [49]	Uganda	NS	226	…	…	…	14	90	47
Nordlander 1990 [50]	Cambodia	>5	50	2	100	…	…	…	70–100
Patwari 1993 [51]	India	>0	533	17	…	…	60	27	90
Sebodo 1978 [52]	Indonesia	NS	92	6	50	…	…	…	75
Venkataraman 2003 [53]	India	>3	262	27	…	…	42	45	79
LMIC adults only or unknown (n = 3)									
Hossain 1991 [15]	Bangladesh	>25	11358	3895	80	74	…	…	…
Pender 2022 [54]	Thailand, Nepal	NS	453	34^c^	…	…	565^c^	85	24
Wang 2014 [55]	China	NS	424	90^c^	…	…	176^c^	99	97
HIC children (n = 11)									
Alvarado 1983 [56]	UK	≥2	376	34	91	…	…	…	82–100
Alzaher 2022 [57]	Saudi Arabia	>0	1985	84	…	…	1766	34	78
Ascher 1991 [58]	USA	>5	180	8	…	…	24	83	96
Caprioli 1996 [59]	Italy	NS	618	2	…	…	168	57–58	73–88
Denno 2005 [60]	USA	>0	226	2	…	…	12	45	96
DuBois 1988 [61]	USA	>2	69	12	83	…	25	82	83
Fan 1993 [62]	USA	NS	1031	20	50	…	55	29	93
Koplan 1980 [63]	USA	NS	27	13	…	…	52	36	69
McIver 2001 [64]	Australia	>0	412	…	…	…	30	43	87
Paccagnini 1987 [13]	Italy	>5	337	…	…	…	62	32–54	90
Park 2019 [31]	South Korea	>0	62	…	…	…	19^c^	44	84
HIC adults only (n = 8)									
Bouckenooghe 2000 [65]	USA	NS	227	…	…	…	56	27	85
Lai 2016 [66]	Taiwan	>1	627	3^c^	…	…	187^c^	45	73
Lever 2021 [67]	UK	NS	1450	25	…	…	269	32	91
Loosli 1985 [68]	Switzerland	NS	119	3	…	…	35	86	67
Miller 1994 [69]	USA	>0	55	9	95	…	…	…	…
Scerpella 1994 [70]	USA	NS	92	32	72	…	36	69	89
Siegel 1987 [71]	USA	≥3	113	19	95	…	54	89	68
Tribble 2008 [72]	USA	>0	182	…	…	…	177	33	85

Abbreviations: HIC, high-income country; HPF, high-power field; LMIC, low- and middle-income country; LPF, low-power field; NS, not specified; UK, United Kingdom; USA, United States; WBC, white blood cell count.

^a^Unless otherwise specified.

^b^Range provided if specificity was calculated based on multiple negative outcome definitions (see Supplementary Table 1 for negative outcome definitions).

^c^Includes detection by polymerase chain reaction; otherwise, detection by culture (see Supplementary Table 1 for details).

**Table 2.** Sensitivity and Specificity of Red Blood Cells or Occult Blood in Stool to Identify *Shigella* and Other Bacterial Causes of Diarrhea in Systematically Reviewed Studies by Setting and Age of Included Individuals (n = 26 Studies)

Setting and Study	Study Location	RBC Test	No. With Diarrhea	No. With *Shigella*	Sensitivity for *Shigella*, %	Specificity for *Shigella*, %	No. With Combined Bacterial Outcome	Sensitivity for Combined Bacterial Outcome, %	Specificity for Combined Bacterial Outcome^a^, %
LMIC children (n = 10)									
Ashraf 2007 [73]	Bangladesh	FOBT	594	18	56	…	73	55	53
Bardhan 2000 [74]	Bangladesh	FOBT	1008	205	87	…	…	…	18–80
Beltinger 1997 [44]	Bangladesh	FOBT	304	38	82	…	54	69	66
Chang 2017 [46]	China	Micro	680	1	…	…	215	13	…
Huicho 1993 [16]	Peru	FOBT	446	10	70	…	58	43	62–69
Huicho 1997 [21]	Peru	FOBT	125	6	…	…	29	79	50
Ismail 1994 [30]	Indonesia	Micro	619	44	68	61	60	52	…
Korzeniowski 1979 [14]	USA, Brazil	FOBT	101	29	85	…	…	…	89
McNeely 1996 [48]	Mexico	FOBT	1040	143	…	…	173	79	64
Patwari 1993 [51]	India	Micro	533	17	…	…	60	32	89
LMIC adults only or unknown (n = 4)									
Pender 2022 [54]	Thailand, Nepal	Micro	453	34^b^	…	…	565^b^	37	85
Wang 2014 [55]	China	Micro	424	90^b^	…	…	176^b^	96	97
Aly 2005 [75]	Egypt	FOBT	40	20	45	…	…	…	30
HIC children (n = 5)									
Alzaher 2022 [57]	Saudi Arabia	FOBT	1985	84	…	…	1766	48	84
Ascher 1991 [58]	USA	FOBT	180	8	…	…	24	37	67
Denno 2005 [60]	USA	FOBT or Micro	226	2	…	…	12	40	96
Paccagnini 1987 [13]	Italy	FOBT	337	…	…	…	62	74–83	67
Park 2019 [31]	South Korea	FOBT	62	…	…	…	19^b^	61	82
HIC adults only (n = 7)									
Bouckenooghe 2000 [65]	USA	FOBT	227	…	…	…	56	30	71
Lai 2016 [66]	Taiwan	FOBT	627	3^b^	…	…	187^b^	53	63
Lever 2021 [67]	UK	Micro	1450	25	…	…	269	16	94
Loosli 1985 [68]	Switzerland	FOBT	119	3	…	…	35	80	66
Scerpella 1994 [70]	USA	FOBT	92	32	37	…	36	37	82
Shastri 2008 [26]	Germany	FOBT	200	2	…	…	107	38	85
Siegel 1987 [71]	USA	FOBT	113	19	84	…	54	87	58
Tribble 2008 [72]	USA	FOBT	182	…	…	…	177	42	84

Abbreviations: FOBT, fecal occult blood test; HIC, high-income country; LMIC, low- and middle-income country; Micro, microscopy; RBC, red blood cell count; UK, United Kingdom; USA, United States.

^a^Range provided if specificity was calculated based on multiple negative outcome definitions (see Supplementary Table 1 for negative outcome definitions).

^b^Includes detection by polymerase chain reaction; otherwise, detection by culture (see Supplementary Table 1 for details).

**Table 3.** Sensitivity and Specificity of Lactoferrin, Calprotectin, or Myeloperoxidase in Stool to Identify *Shigella* and Other Bacterial Causes of Diarrhea in Systematically Reviewed Studies (n = 19 Studies)

Setting and Study	Study Location	Biomarker Cutoff	No. With Diarrhea	No. With *Shigella*	Sensitivity for *Shigella*, %	Specificity for *Shigella*, %	No. With Combined Bacterial Outcome	Sensitivity for Combined Bacterial Outcome, %	Specificity for Combined Bacterial Outcome, %
Lactoferrin (n = 13)									
Aly 2005 [75]	Egypt	1:50	40	20	80	…	…	…	25
Ashraf 2007 [73]	Bangladesh	1:50	594	18	61	…	73	52	68
Bouckenooghe 2000 [65]	USA	1:50	227	…	…	…	56	27	79
Choi 1996 [20]	USA	1:50	46	3	…	…	28	93	83
Huicho 1997 [21]	Peru	1:50	125	6	…	…	29	97	15
McIver 2001 [64]	Australia	NS	412	…	…	…	30	95	40
Mercado 2011 [18]	Peru	NS	935	…	…	…	200	95	…
Miller 1994 [69]	USA	1:50	55	9	100	…	43	60	8
Park 2019 [31]	South Korea	22.8 µg/mL	62	…	…	…	19^a^	78	71
Scerpella 1994 [70]	USA	1:50	92	32	94	…	36	94	47
Shastri 2008 [26]	Germany	1:400	200	2	…	…	107	78	54
Tribble 2008 [72]	USA	1:50	182	…	…	…	177	93	56
Venkataraman 2003 [53]	India	1:50	262	27	…	…	42	83	28
Calprotectin (n = 8)									
Ahn 2020 [76]	South Korea	388 mg/kg	400	7^a^	…	…	197^a^	71	61
Berger 2010 [77]	Unknown	>50 µg/mL	168	…	…	…	108	80	89
Czub 2014 [78]	Poland	15 µg/mL	50	…	…	…	21	100	45
Duman 2015 [23]	Turkey	710 mg/L	84	7	78	…	9	89	76
Kim 2022 [79]	South Korea	815 µg/g	80	…	…	…	16^a^	75^b^	40
Park 2019 [31]	South Korea	74.0 µg/g	62	…	…	…	19^a^	94	39
Shastri 2008 [26]	Germany	14.9 mg/L	200	2	…	…	107	83	87
Sýkora 2010 [27]	Czech Republic	103.9 µg/g	66	…	…	…	31	93	88
Myeloperoxidase (n = 1)									
Park 2019 [31]	South Korea	4.14 ng/mL	62	…	…	…	19^a^	78	57

Abbreviations: NS, not specified; USA, United States.

^a^Includes detection by polymerase chain reaction; otherwise, detection by culture (see Supplementary Table 1 for details).

^b^Estimated from manuscript figure.

Different definitions of positive for WBC by microscopy (ranging from >0 to >20 cells per high-power field) and different assays and/or cutoffs for the other biomarkers made it difficult to compare results across studies. Similarly, heterogeneity resulted from differences in the combined bacterial outcome considered and the negative outcome used to calculate specificity. Most studies (n = 43 [88%]) were considered low quality (Supplementary Table 2) due to using bacterial culture, which is an insensitive gold standard diagnostic, particularly for *Shigella* [7]. Only 12 studies (24%) were conducted among children in LMICs (ie, the target population for EFGH), none of which used molecular diagnostics and only 2 of which assessed either lactoferrin or calprotectin. Only 6 (12%) studies included >1000 individuals with diarrhea.

## METHODS IN THE EFGH INFLAMMATORY BIOMARKER SUBSTUDY

We will conduct an inflammatory biomarker substudy in 6 EFGH sites: Bangladesh, Kenya, Malawi, Pakistan, Peru, and The Gambia. The objective of this substudy is to evaluate whether inflammatory biomarkers measured in whole stool can identify the bacterial subset of diarrhea episodes, and shigellosis specifically. The primary EFGH study design is described elsewhere [32].

### Sample Collection

Whole stool samples will be collected as soon as possible after enrollment from all enrolled children aged 6–35 months presenting with diarrhea at selected study health facilities. Samples will be collected if they are produced at any time while the participant is present at the enrolling facility or within 24 hours of leaving the enrolling facility. This strategy will increase the yield of whole stool collections since children may not produce stool during the enrollment visit. Study staff will conduct home visits to collect stools produced after leaving the facility. Caregivers will also have the option of returning the whole stool sample to the enrollment facility. In both cases, caregivers of participants who do not produce whole stool at the enrollment visit will be provided with a whole stool collection kit and will be instructed to collect the participant's first stool produced after leaving the enrollment facility. Home visits will only occur during routine working hours. Once retrieved by study personnel (and within 18 hours of stool production), whole stool samples will be placed into a cool box (2°C–8°C) for transportation to the laboratory. The following will be verified during accession: labeling, stool volume, and transport conditions, which include packaging and temperature monitoring using WarmMark (after collection by staff only). Whole stool will be aliquoted into up to five 2-mL cryotubes (up to 1 g per cryotube), and frozen at −80°C until testing.

### Inflammatory Biomarker Testing

All whole stool samples will be tested for 4 biomarkers: MPO, calprotectin, lipocalin-2 (NGAL), and hemoglobin. These 4 were chosen to capture markers of both leukocytes and erythrocytes since both showed evidence of diagnostic ability in the systematic review. While most prior studies used microscopy to measure fecal WBC and RBC, microscopy would be impractical as a diagnostic in many settings; therefore, we selected protein biomarkers of leukocytes and erythrocytes that either had prior evidence of diagnostic ability (calprotectin, hemoglobin), had strong preliminary data from our prior work (MPO), or represented a novel component of the host immune response, which would limit collinearity between markers (NGAL). Lactoferrin was considered but ultimately rejected given its similarity with calprotectin and the better sensitivity for calprotectin over lactoferrin in studies from the systematic review that assessed both [26, 31]. We will not evaluate systemic biomarkers given the likely infeasibility and unacceptability of collecting blood samples at the POC in most clinical settings.

Each marker, their underlying mechanism of action, and rationale are outlined in Table 4. We will use commercially available enzyme-linked immunosorbent assays (ELISAs) for each biomarker according to their manufacturers’ instruction manuals [33–36]. Biomarker concentrations per gram of stool will be calculated from the raw optical density data using a 4-parameter curve fit to the standards, which will be run in duplicate on every plate. Each plate will also include a high and low concentration control run in duplicate. The analysis of raw optical densities will be centralized using a custom-built R-based Shiny application accessible on the web. The app will allow for monitoring of standards and controls with immediate feedback to the laboratories at each site and real-time quality control (QC) monitoring by a central coordinating team.

**Table 4.** Fecal Inflammatory Biomarkers to Be Assessed in the Enterics for Global Health Study

Biomarker	Target	Description	Rationale
Myeloperoxidase	Neutrophils	A peroxidase enzyme belonging to the heme-containing proteins, produced largely in neutrophils and in smaller quantities in monocytes [80, 81]. MPO is considered a biomarker for neutrophils, inflammatory activity in the gastrointestinal tract, and neutrophil damage [81]. Fecal MPO is a biomarker for IBD [82].	*Shigella* detections in MAL-ED were associated with increases in MPO, and the association depended on *Shigella* quantity, such that MPO levels were more highly elevated as the quantity of *Shigella* increased [10]. Diarrheagenic *Escherichia coli*, specifically EAEC, EPEC, and ETEC, was also associated with elevated MPO, though to a lesser extent [83].
Calprotectin	Neutrophils	A protein biomarker of leukocytes and neutrophil damage during intestinal inflammation [23–25]. Fecal calprotectin helps to distinguish between IBD and noninflammatory bowel conditions and monitor IBD activity [84].	Increased levels were observed among those with bacterial compared to viral infections during diarrhea [24]. Calprotectin was elevated in shigellosis at levels higher than other bacterial diarrheas (*Clostridioides difficile*, *Salmonella*, *Campylobacter*, and EIEC) [85].
Lipocalin-2 (NGAL)	Neutrophils; enterocyte damage	A circulatory protein commonly referred to as neutrophil gelatinase-associated lipocalin (NGAL). NGAL is responsible for the delivery of molecules including steroids, free fatty acids, and hormones to body organs [86]. It is an indicator of innate immunity [87], found in a variety of cells including neutrophils, and possesses antibacterial and anti-inflammatory functions, in addition to providing protection against cell and tissue stress [86]. NGAL is an indicator of enterocyte damage and acute and chronic renal injury [86, 88]. It is also a biomarker for intestinal inflammation and is associated with IBD [87].	Studies in a *Shigella* murine model demonstrate that sensitivity for *Shigella* may be higher compared to MPO [89]. Lipocalin-2 decreases rapidly following inflammation [90].
Hemoglobin	Fecal occult blood	The iron-containing protein present in RBCs responsible for transporting oxygen to organs and tissues [91]. Hemoglobin is a marker of RBCs and its presence in stool indicates the presence of blood. Fecal hemoglobin helps to identify IBD patients with active inflammation [92].	*Shigella* is the main cause of dysentery among children globally [7]. Presence of RBCs was predictive of shigellosis and other bacterial diarrhea in the systematic review.

Abbreviations: EAEC, enteroaggregative *Escherichia coli*; EIEC, enteroinvasive *Escherichia coli*; EPEC, enteropathogenic *Escherichia coli*; ETEC, enterotoxigenic *Escherichia coli*; IBD, inflammatory bowel disease; MAL-ED, Etiology, Risk Factors, and Interactions of Enteric Infections and Malnutrition and the Consequences for Child Health and Development; MPO, myeloperoxidase; NGAL, neutrophil gelatinase-associated lipocalin; RBCs, red blood cells.

### Detection of Enteric Pathogens

Total nucleic acid will be extracted from rectal swabs from each enrolled participant and tested for enteric pathogens by quantitative PCR (qPCR) using the TaqMan Array Card platform, as described elsewhere in this supplement [37]. Attribution of diarrhea episodes to specific infectious etiologies will be based on the quantities of pathogens detected, and assigned etiologies by qPCR will be considered the “gold standard” diagnostic against which we will compare the inflammatory biomarkers. Specifically, attribution will be assigned if a pathogen is detected at a cycle threshold value below the EFGH and pathogen-specific cutoff, described elsewhere [37]. The definition of etiology will not be dependent on the detection of other pathogens, such that multiple etiologies may be identified for each episode. The presence of co-etiologies will not be considered in the primary analysis (eg, if rotavirus is considered etiologic in addition to *Shigella*), but we will exclude episodes with multiple etiologies in a sensitivity analysis. *Shigella* will also be identified by bacterial culture in the main EFGH study, which will be considered in a separate sensitivity analysis.

### Data Analysis

We will compare the diagnostic characteristics (eg, area under the curve, sensitivity, specificity) of the candidate biomarkers to identify watery diarrhea episodes that are attributed to *Shigella*, specific *Shigella* species, and other causes of bacterial diarrhea (eg, typical EPEC, ETEC, *Campylobacter, Salmonella,* and *V cholerae*) by qPCR. The correlation between markers will also be assessed to determine which may be complementary and/or redundant. Clinical prediction scores will be derived that incorporate the best-performing inflammatory biomarkers to identify an optimal diagnostic tool for watery bacterial diarrhea, and *Shigella*-attributed cases in particular. We will use SuperLearner, an algorithm that uses cross-validation to create an “ensemble” prediction model, which is an optimal weighted average of multiple machine learning models. Clinical and epidemiologic characteristics will include fever, duration of diarrhea, dehydration, vomiting, stool frequency, child age, season, length-for-age *z* score (LAZ, if <24 months of age) or height-for-age *z* score (HAZ, if ≥24 months of age), and breastfeeding at enrollment. Based on the variables included in the ensemble prediction model, we will create a more parsimonious prediction score based on scaled coefficients from a logistic regression model for bacterial etiology. This score could be practically applied in clinical settings; each characteristic included will be assigned points that would then be summed into a total score. Acknowledging the need to prioritize specificity to limit antibiotic overuse, we will derive cutoffs that maximize sensitivity at a minimum level of specificity of 80% to identify episodes that should be treated according to our algorithm. We will also report categories of confidence (eg, “most likely bacterial”) and/or percentage confidence based on the optimal machine learning algorithm. Finally, based on antibiotic treatments received and antibiotic susceptibility data [38], we will estimate the impact of appropriate antibiotic treatment on duration of diarrhea, hospitalization, 90-day mortality, and change in HAZ/LAZ in the subset of diarrhea episodes meeting the threshold for treatment based on the optimal treatment algorithm. We will compare the effects of treatment in the algorithm-defined subset with those among all episodes, in the subset of episodes that would be treated according to World Health Organization (WHO) guidelines (ie, dysentery only), and in etiology-specific subsets based on pathogen quantity detected by qPCR.

Preliminary analyses of MAL-ED data suggest that the biomarkers will be successful in identifying bacterial diarrhea. We added MPO concentrations that were de-trended for age to a clinical prediction score for *Shigella* previously developed in MAL-ED [7] to assess improvements in predictive ability of the score. The best improvements were achieved when MPO concentration was included with 5 categories and was weighed similarly heavily as child age. The clinical score alone achieved 40% sensitivity for identifying *Shigella*-attributable diarrhea episodes with 80% specificity (AUC = 0.74) in the subset of episodes that were also tested for MPO (n = 281). The addition of MPO increased sensitivity to 70% with 80% specificity (AUC = 0.79). This is a substantial improvement that may be more striking with the addition of multiple candidate biomarkers in a larger dataset.

## POTENTIAL CHALLENGES AND LIMITATIONS

Our approach has some noteworthy limitations. Foremost, the algorithm will likely identify at least some episodes that should not be treated with antibiotics. Specifically, it could lead to unnecessary treatment of children with viral or parasitic diarrhea, which would facilitate antibiotic overuse and have implications for the development of antimicrobial resistance. However, use of even an imperfectly specific algorithm to inform treatment decisions would likely improve on current clinical practice given the often extreme overuse of antibiotics for diarrhea among children in low-resource settings [39]. The algorithm could also in theory cause harm by identifying bacterial diarrhea episodes for which antibiotics are contraindicated, for example, for children with Shiga toxin–producing *E coli* (STEC) [40]. However, the typical STEC clinical syndrome is bloody diarrhea without fever, whereas this algorithm would be primarily relevant for watery diarrhea since the WHO guidelines already recommend treatment for dysentery [6]. Furthermore, there was weak association between STEC and diarrhea in GEMS and MAL-ED, such that the role of STEC in children in resource-limited settings may be limited [7, 41]. Another key limitation is that the biomarker tests will not provide antibiotic susceptibility results, such that the algorithm will not be able to determine which antibiotic is likely to be effective in cases in which antibiotics are indicated. Clinicians will have to continue to rely on any available local susceptibility data.

There will also be several challenges in the research methods. Because not all participants will produce a stool specimen at the clinic during the enrollment visit, we will include stool samples produced within 24 hours of leaving the enrollment facility to achieve the sample size required to adequately power the study. This may confound the results in 2 ways. First, sampling will be performed at variable time points from the onset of illness, which may impact the levels of biomarkers within stool. Second, since antibiotics are likely to be administered before and/or at the enrollment visit, the biomarker levels in stools may have changed as a result of antibiotic action. We will adjust for the time between presentation to care and sample collection in the analysis to mitigate this concern. We will evaluate whether the performance of the algorithm differs by time since symptom onset and antibiotics received, since there will also be heterogeneity in these factors at the POC.

The inclusion of stool samples collected at participants’ homes may also result in variable time in which samples are outside of cold chain. While NGAL, calprotectin, and MPO are stable at room temperature [34–36], hemoglobin degrades up to 50% per day at room temperature [33]. In addition, to maximize the efficiency of sample testing, stool samples may be stored frozen for variable amounts of time and up to several months before being tested. While the test manufacturers stipulate that stool samples for the NGAL and calprotectin assays can be stored at −20°C for 1 year, samples for the hemoglobin and MPO assays should not be stored at −20°C for more than 1 and 2.5 months, respectively [33–36]. We will test all samples regardless and adjust for storage time in the analysis as necessary.

Next, the quantitative biomarker assays employed in this study require 15–50 mg of whole stool. The gold standard diagnostic comparator (pathogen detection by qPCR) will be evaluated in rectal swabs collected during the enrollment visit, rather than in the same whole stool sample in which the biomarkers will be tested. This aligns with the parent study protocol, which specifies using rectal swabs for qPCR to ensure etiology information is available for every enrolled case. Biomarker concentrations and/or pathogen detection may differ between the 2 samples due to differences in sample type and time of collection. While some patients and providers may prefer collection of whole stool rather than rectal swabs, whole stool may be an impractical clinical specimen on which to base a POC test for bacterial diarrhea since a rapid result would be required to guide clinical management. Should this study show that biomarkers are useful for predicting which children have bacterial diarrhea, future work will need to establish the validity of rectal swabs for POC biomarker testing.

A strength of our study is the inclusion of all medically attended diarrhea cases with etiology determined by qPCR among children from a diverse range of geographical locations. The inclusion of less-severe diarrhea is important since mortality is likely to be similar in both moderate-to-severe and less-severe cases [42]. The inclusion of 6 different study locations, however, presents a challenge when ensuring standardization of laboratory procedures between multiple sites. To ensure that the results are reproducible between sites, standardized ELISA training is being performed at all study sites and the centralized analysis platform will facilitate QC monitoring. In addition, there may be regional differences in the baseline levels of inflammatory biomarkers between populations driven by population genetics, the microbiota, subclinical infections, and/or differences in diet. If large enough, these differences may mean that generalizable biomarker concentration cutoff levels capable of guiding bacterial diagnosis cannot be established. We will evaluate and describe heterogeneity in the performance of the algorithm by study site.

## DISCUSSION

Despite the limitations of the included studies in the systematic review, the sensitivity of all biomarkers to identify *Shigella* was high, and sensitivity to identify combined bacterial outcomes was moderate. Not surprisingly, specificity was lower for *Shigella* than for combined bacterial outcomes since multiple causes of diarrhea are inflammatory. The insensitivity of culture for *Shigella* may also have resulted in lower estimated specificities for *Shigella.* Performance was broadly comparable across biomarkers, justifying the assessment of multiple markers of leukocytes and erythrocytes in EFGH.

Our proposed inflammatory biomarker substudy will improve on prior efforts to use fecal biomarkers to identify bacterial diarrhea by applying ELISAs and molecular diagnostics in a large, geographically diverse study population. By combining inflammatory biomarker test results with clinical prediction scores to maximize predictive validity, we will determine whether POC biomarker tests would be a viable strategy to improve appropriate antibiotic treatment of watery bacterial diarrhea episodes. In the absence of readily available diagnostics for enteric pathogens, these tools could improve short- and long-term outcomes of diarrhea. If the inflammatory biomarkers are acceptably sensitive and specific, further development of low-cost POC biomarker tests would be warranted. Conversely, if these tools are not adequate, development of low-cost assays for the direct detection of enteropathogens in the clinical setting should be prioritized. Either type of POC test would have the dual benefit of increasing appropriate treatment of the episodes that are likely to respond, while also reducing inappropriate and overtreatment of viral and parasitic episodes. In settings where antibiotic overuse is common, the application of such tests may improve access to appropriate therapy while decreasing antibiotic use for diarrhea overall.

## Supplementary Data

Supplementary materials are available at *Open Forum Infectious Diseases* online. Consisting of data provided by the authors to benefit the reader, the posted materials are not copyedited and are the sole responsibility of the authors, so questions or comments should be addressed to the corresponding author.

## Supplementary Material

ofad652_Supplementary_Data

## Notes

***Author contributions.*** C. B., H. B., T. R. B., J. C., S. Q., C. S., W. V. S. L., K. C. J., M. N. K., S. M., U. N. I., F. N. Q., F. Q., and E. T. R. M. actively participated in monthly working group meetings during which the conceptualization and outline were discussed and agreed upon. C. B., H. B., W. V. S. L., S. Q., and E. T. R. M. conducted the systematic review. C. B., H. B., T. R. B., J. C., S. Q., C. S., W. V. S. L., and E. T. R. M. wrote the first draft of the manuscript. K. C. J., M. N. K., S. M., U. N. I., F. N. Q., and F. Q. provided review, scientific input, and editing of the first draft of the manuscript. M. A., H. E. A., K. B., S. A. B., B. E. C., A. K. C., N. A. C., P. F. G. B., S. H., B. H., M. J. H., J. I., T. I., S. I., F. K., K. L. K., T. M., K. M. V., G. M. M., V. M., J. B. O., B. O., M. P. O., R. O., P. B. P., J. A. P.-M., K. T. S., O. S., S. M. T., P. P. Y., and M. T. Y. reviewed and edited the manuscript. All authors approved the content of the final manuscript.

***Disclaimer.*** The views expressed are those of the author(s) and not necessarily those of the National Institute for Health and Care Research (NIHR), the Department of Health and Social Care, the UK government, or the UK Health Security Agency.

***Financial support.*** This project is supported by the Bill & Melinda Gates Foundation (grant numbers INV-016650, INV-031791, INV-036891, INV-036892, INV-028721, INV-041730, INV-044311, INV-044317) and the US National Institutes of Health (grant number D43TW010913 to M. N. K. and M. P. O.). The Gambia team's work is also supported by the UK Research and Innovation Medical Research Council (program number MC_UU_00031—Disease Control and Elimination). N. A. C. is an NIHR Senior Investigator (NIHR203756). N. A. C., J. C., and K. C. J. are affiliated with the NIHR Global Health Research Group on Gastrointestinal Infections at the University of Liverpool, and with the NIHR Health Protection Research Unit in Gastrointestinal Infections at the University of Liverpool, a partnership with the UK Health Security Agency in collaboration with the University of Warwick.

***Supplement sponsorship***. This article appears as part of the supplement “Enterics for Global Health (EFGH) *Shigella* Surveillance Study-Rationale and Methods,” sponsored by the Bill & Melinda Gates Foundation.
