# Supplementary material for: Evaluation of Fecal Inflammatory Biomarkers to Identify Bacterial Diarrhea Episodes: Systematic Review and Protocol for the Enterics for Global Health Shigella Surveillance Study
Source: Open Forum Infect Dis. 2024 Mar 25;11(Suppl 1):S65–75. doi: 10.1093/ofid/ofad652 (PMC10962755; doi:10.1093/ofid/ofad652)
Supplement: ofad652_Supplementary_Data [file ofad652_supplementary_data.docx]

**Appendix**

Evaluation of fecal inflammatory biomarkers to identify bacterial diarrhea episodes: Systematic review and Protocol for the Enterics for Global Health – *Shigella* Surveillance Study

**Systematic review search terms**

*PubMed (searched on 04/03/2023):*

(diarrh*[Title/Abstract] OR acute gastroenteritis[Title/Abstract] OR shigell*[Title/Abstract] OR campylobacter* [Title/Abstract] OR enterotoxigenic Escherichia coli[Title/Abstract] OR ETEC[Title/Abstract] OR enteropathogenic escherichia coli[Title/Abstract] OR enteropathogenic e. coli[Title/Abstract] OR EPEC[Title/Abstract] OR Shiga toxin producing Escherichia coli[Title/Abstract] OR Shiga toxin-producing E. coli[Title/Abstract] OR STEC [Title/Abstract] OR enterohemorrhagic Escherichia coli[Title/Abstract] OR enterohemorrhagic e. coli [Title/Abstract] OR EHEC[Title/Abstract] OR enteroinvasive Escherichia coli [Title/Abstract] OR enteroinvasive E. coli[Title/Abstract] OR EIEC[Title/Abstract] OR dysentery[Title/Abstract] OR cholera*[Title/Abstract] OR vibrio cholera*[Title/Abstract] OR bacillary[Title/Abstract]) AND (child* OR adult* OR human OR patient*) AND (leukocyte*[Title/Abstract] OR leucocyte*[Title/Abstract] OR fecal leukocyte*[Title/Abstract] OR fecal leucocyte*[Title/Abstract] OR erythrocyte*[Title/Abstract] OR white blood cell*[Title/Abstract] OR red blood cell*[Title/Abstract] OR lipocalin*[Title/Abstract] OR calprotectin[Title/Abstract] OR myeloperoxidase[Title/Abstract] OR pentraxin*[Title/Abstract] OR lactoferrin[Title/Abstract] OR hemoglobin[Title/Abstract] OR haemoglobin[Title/Abstract] OR mpo[Title/Abstract] OR wbc[Title/Abstract] OR rbc[Title/Abstract] OR occult blood[Title/Abstract]) AND (fecal test[Title/Abstract] OR faecal test[Title/Abstract] OR diagnos*[Title/Abstract] OR clinical prediction[Title/Abstract] OR point-of-care[Title/Abstract] OR point of care[Title/Abstract] OR biomarker[Title/Abstract] OR marker[Title/Abstract] OR screening[Title/Abstract] OR sensitiv*[Title/Abstract] OR specificity[Title/Abstract] OR fecal microscopy[Title/Abstract] OR faecal microscopy[Title/Abstract] OR fecal occult[Title/Abstract] OR faecal occult[Title/Abstract] OR occult blood[Title/Abstract] OR stool test[Title/Abstract] OR identification[Title/Abstract] OR compare[Title/Abstract] OR probability[Title/Abstract] OR predict*[Title/Abstract])

*Embase (searched on 05/01/2023):*

(diarrh*:ab,ti OR 'acute gastroenteritis':ab,ti OR shigell*:ab,ti OR campylobacter*:ab,ti OR 'enterotoxigenic escherichia coli':ab,ti OR etec:ab,ti OR 'enteropathogenic escherichia coli':ab,ti OR 'enteropathogenic e. coli':ab,ti OR epec:ab,ti OR 'shiga toxin producing escherichia coli':ab,ti OR 'shiga toxin-producing e. coli':ab,ti OR stec:ab,ti OR 'enterohemorrhagic escherichia coli':ab,ti OR 'enterohemorrhagic e. coli':ab,ti OR ehec:ab,ti OR 'enteroinvasive escherichia coli':ab,ti OR 'enteroinvasive e. coli':ab,ti OR eiec:ab,ti OR dysentery:ab,ti OR cholera*:ab,ti OR 'vibrio cholera*':ab,ti OR bacillary:ab,ti) AND (child* OR adult* OR human OR patient*) AND (leukocyte*:ab,ti OR leucocyte*:ab,ti OR 'fecal leukocyte*':ab,ti OR 'fecal leucocyte*':ab,ti OR erythrocyte*:ab,ti OR 'white blood cell*':ab,ti OR 'red blood cell*':ab,ti OR lipocalin*:ab,ti OR calprotectin:ab,ti OR myeloperoxidase:ab,ti OR pentraxin*:ab,ti OR lactoferrin:ab,ti OR hemoglobin:ab,ti OR haemoglobin:ab,ti OR mpo:ab,ti OR wbc:ab,ti OR rbc:ab,ti OR 'occult blood':ab,ti) AND ('fecal test':ab,ti OR 'faecal testor diagnos*':ab,ti OR 'clinical prediction':ab,ti OR 'point of care':ab,ti OR biomarker:ab,ti OR marker:ab,ti OR screening:ab,ti OR sensitiv*:ab,ti OR specificity:ab,ti OR 'fecal microscopy':ab,ti OR 'faecal microscopy':ab,ti OR 'fecal occult':ab,ti OR 'faecal occult':ab,ti OR 'occult blood':ab,ti OR 'stool test':ab,ti OR identification:ab,ti OR compare:ab,ti OR probability:ab,ti OR predict*:ab,ti)

Studies included in review **(n = 49)**

Studies excluded **(n = 3782)**

Studies not retrieved **(n = 0)**

Studies assessed for eligibility **(n = 93)**

Studies sought for retrieval **(n = 93)**

Studies screened **(n = 3875)**

Studies excluded **(n = 44)**

Publication date prior to 1970 (n = 2)

Not available in English (n = 11)

Review article (n = 5)

Natural history study (n = 2)

Ineligible biomarker assessed (n = 4)

Ineligible outcome assessed (n = 10)

Insufficient data reported (n = 10)

Studies from databases/registers **(n = 5022)**

Embase (n = 2864)

PubMed (n = 2158)

**Identification**

Duplicates removed **(n = 1147)**

**Screening**

**Included**

Appendix Figure 1. PRISMA flowchart of studies in the systematic review.

Appendix Table 1. Details of included studies evaluating the sensitivity and specificity of inflammatory biomarkers for the identification of bacterial diarrhea in the systematic review.

|  | **Enrollment Dates** | **Study Location** | **Study**  **Population** | **Gold Standard**  **Diagnostic** | **Combined Bacterial Outcome** | **Negative Outcome(s) for**  **Specificity Calculation*** |
| --- | --- | --- | --- | --- | --- | --- |
| Ahn 2020 [1] | 2016-2018 | South Korea | >18 years | PCR | All bacteria | No bacteria |
| Alvarado 1983 [2] | 1981 | United Kingdom | 0-85 years | Culture | NA | No pathogen, viruses, parasites |
| Aly 2005 [3] | NS | Egypt | NS | Culture | NA | *E. histolytica* |
| Alzaher 2022 [4] | 2008-2020 | Kingdom of Saudi Arabia | All ages | Culture | All bacteria | No bacteria |
| Ascher 1991 [5] | 1986 | United States | 0-6 years | Culture | All bacteria | No bacteria |
| Ashraf 2007 [6] | 2002 | Bangladesh | 0-80 years | Culture | Inflammatory pathogens (*Shigella*, *Salmonella*, *Campylobacter*, STEC, EIEC) | No pathogen |
| Bardhan 2000 [7] | NS | Bangladesh | All ages | Culture | NA | No pathogen, *E. histolytica*, rotavirus |
| Beltinger 1997 [8] | 1990 | Bangladesh | All ages | Culture | *Shigella*, *Salmonella* | Non-inflammatory pathogens (*Vibrio* and no pathogens) |
| Berger 2010 [9] | 2008 | NS | NS | Culture | All bacteria | No bacteria |
| Bodhidatta 2002 [10] | 1998-2000 | Thailand | <12 years | Culture | NA | NA |
| Bouckenooghe 2000 [11] | 1997-1998 | United States | Adult travelers to Guadalajara Mexico or Goa, India | Culture | ETEC | No pathogen |
| Caprioli 1996 [12] | 1992 | Italy | <10 years | Culture | *Campylobacter*, *Salmonella* | Rotavirus, adenovirus, no pathogen |
| Chang 201 7[13] | 2014 | China | All ages | Culture | All bacteria | No bacteria |
| Choi 1996 [14] | 1994-1995 | United States | 2-84 years | Culture | All bacteria | No bacteria |
| Czub 2014 [15] | NS | Poland | 1-8 years | Culture | *Salmonella* | Rotavirus |
| Denno 2005 [16] | 1998-2001 | United States | <21 years | Culture | All bacteria | No bacteria |
| DuBois 1988 [17] | NS | United States | All ages | Culture | All bacteria | No bacteria |
| Duman 2015 [18] | 2009-2018 | Turkey | <18 years | Culture | All bacteria | No bacteria |
| Fan 1993 [19] | 1991 | United States | All ages | Culture | *Shigella*, *Campylobacter*, *Salmonella* | No bacteria |
| Hossain 1991 [20] | 1977-1981 | Bangladesh | NS | Culture | NA | No *Shigella* |
| Huicho 1993 [21] | 1986-1989 | Peru | 0-2 years | Culture | Invasive bacteria (*Shigella*, *Campylobacter*, *Salmonella*) | No pathogen, *Cryptosporidium*, rotavirus, parasites |
| Huicho 1997 [22] | 1995-1996 | Peru | <2 years | Culture | *Shigella*, *Campylobacter*, *Salmonella* | No bacteria |
| Ismail 1994 [23] | 1991-1992 | Indonesia | 0-5 years | Culture | Invasive bacteria (*Shigella*, *Salmonella*, *Campylobacter*, *Aeromonas*, *Plesiomonas*) | No invasive bacteria |
| Jindal 1991 [24] | NS | India | 0-1 years | Culture | All bacteria | No bacteria |
| Khan 2006 [25] | 2000-2001 | Bangladesh | All ages | Culture | NA | No *Shigella* |
| Kim 2022 [26] | 2015-2020 | South Korea | 1-18 years | PCR | All bacteria | No bacteria |
| Koplan 1980 [27] | 1997 | United States | All ages | Culture | All bacteria | No bacteria |
| Korzeniowski 1979 [28] | NS | United States; Brazil | 0-74 years | Culture | NA | No bacteria |
| Lai 2016 [29] | 2005-2009 | Taiwan | ≥15 years | Culture and PCR | All bacteria | No bacteria |
| Lever 2021 [30] | 2010 & 2016 | United Kingdom | ≥16 years | Culture | All bacteria | Parasites |
| Loosli 1985 [31] | 1981-1982 | Switzerland | >15 years | Culture | Invasive bacteria (*Shigella, Salmonella, Campylobacter*) | No invasive bacteria |
| McIver 2001 [32] | 1997-1998 | Australia | <6 years | Culture | *Salmonella*, *Campylobacter* | No bacteria |
| McNeely 1996 [33] | 1985; 1988-1992 | Mexico | American college students in Mexico and Mexican children: 0-7 years | Culture | Invasive bacteria (*Shigella*, *Salmonella*, *Campylobacter*) | No invasive bacteria |
| Mercado 2011 [34] | 2006-2007; 2008 | Peru | 0-2 years | Culture | For WBC results: EAEC, EPEC, ETEC, DAEC, STEC, *Campylobacter;* For lactoferrin results: EAEC, EPEC, ETEC, DAEC, STEC | No pathogen |
| Miller 1994 [35] | 1991 & 1992 | United States | 18-40 years | Culture | *Shigella*, *V. cholerae,* EPEC, ETEC | Nosocomial *C. difficile* |
| Mshana 2010 [36] | 2005 | Uganda | Children | Culture | *Campylobacter* | No *Campylobacter* |
| Nordlander 1990 [37] | 1989 | Cambodia | <2 years | Culture | NA | Rotavirus, no pathogen, *Cryptosporidium*, *Giardia* |
| Paccagnini 1987 [38] | 1984-1986 | Italy | Children | Culture | *Campylobacter*, *Salmonella* | No bacteria |
| Park 2019 [39] | 2013-2014 | South Korea | 0-18 years | Culture and PCR | All bacteria | No bacteria |
| Patwari 1993 [40] | 1987-1990 | India | <5 years | Culture | Invasive bacteria (*Shigella*, *Salmonella,* EIEC) | No invasive bacteria |
| Pender 2022 [41] | 2012-2014; 2016-2017 | Thailand; Nepal | ≥18 years | Culture and PCR | All bacteria | No bacteria |
| Scerpella 1994 [42] | 1988 | United States | Adult travelers to Mexico | Culture | *Shigella*, *Salmonella*, *Campylobacter* | No bacteria |
| Sebodo 1978 [43] | 1975 | Indonesia | <3 years | Culture | NA | No bacteria |
| Shastri 2008 [44] | 2004-2007 | Germany | ≥18 years | Culture | All bacteria | No bacteria |
| Siegel 1987 [45] | 1984-1986 | United States | ≥18 years | Culture | All bacteria | No bacteria |
| Sykora 2010 [46] | NS | Czech Republic | 0-3 years | Culture | All bacteria | No bacteria |
| Tribble 2008 [47] | 2000-2001 | United States | Military personnel in Thailand | Culture | Invasive pathogens (*Campylobacter*, *Salmonella*, *Plesiomonas*) | No invasive pathogens |
| Venkataraman 2003 [48] | NS | India | <12 years | Culture | Invasive pathogens (*Shigella*, *Campylobacter*,  *Salmonella*) | No invasive pathogens |
| Wang 2014 [49] | 2006-2008 | China | ≥18 years | PCR | All bacteria | Non-infectious diarrhea |

NS: Not specified; NA: Not applicable; WBC: white blood cell; STEC: Shiga toxin-producing *Escherichia coli*; EIEC: enteroinvasive *E. coli*; ETEC: enterotoxigenic *E. coli*; EPEC: enteropathogenic *E. coli*; EAEC: enteroaggregative *E. coli*; DAEC: diffusely adherent *E. coli*

*If multiple negative outcomes were used to calculate specificities, a range of specificities was reported in the main tables

Appendix Table 2. Summary of Quality Assessment of Diagnostic Accuracy Studies (QUADAS) criteria (see specific operationalized definitions in footnotes) for studies evaluating the sensitivity and specificity of inflammatory biomarkers for the identification of bacterial diarrhea included in the systematic review.

| Citation | Representative patients^1^ | Clear selection criteria^2^ | Accurate reference standard^3^ | Appropriate time-period^4^ | Universal application of reference test^5^ | Received same reference test despite index result^6^ | Index not part of reference^7^ | Index test defined^8^ | Reference test defined^9^ | Index interpret without reference^10^ | Reference interpret without index^11^ | Clinical data available^12^ | Uninterpretable results reported^13^ | Withdrawal explained^14^ |
| --- | --- | --- | --- | --- | --- | --- | --- | --- | --- | --- | --- | --- | --- | --- |
| Ahn 2020 [1] | N | Y | Y | Y | Y | Y | Y | Y | Y | Un | Un | Y | Y | N |
| Alvarado 1983 [2] | N | Y | N | Y | Y | Un | Y | Y | N | Y | Un | Y | Y | Y |
| Aly 2005 [3] | N | N | N | Un | Y | Y | Y | Y | Y | N | Y | Y | Y | Y |
| Alzaher 2022 [4] | N | Y | N | Y | N | Y | Y | N | Y | Un | Un | Y | Y | Y |
| Ascher 1991 [5] | N | Y | N | Y | Y | Y | Y | N | Y | Un | Un | Y | Y | Y |
| Ashraf 2007 [6] | N | Y | N | Un | Y | Y | Y | Y | Y | Un | Un | Y | Y | Y |
| Bardhan 2000 [7] | Y | N | N | Un | Y | Y | Y | Y | Y | Y | Y | Y | Y | Y |
| Beltinger 1997 [8] | Y | Y | N | Un | Y | Y | Y | Y | Y | Y | Y | Y | Y | Y |
| Berger 2010 [9] | Un | N | N | Un | Y | Y | Y | N | N | Un | Un | Y | Y | Un |
| Bodhidatta 2002 [10] | N | Y | N | Un | Y | Y | Y | N | Y | Y | Un | Y | Y | Y |
| Bouckenooghe 2000 [11] | N | Y | N | Y | Y | Y | Y | Y | Y | Un | Un | Y | Y | Y |
| Caprioli 1996 [12] | N | Y | N | Y | Y | Y | Y | Y | Y | Y | Un | Y | Y | Y |
| Chang 201 7[13] | Y | Y | N | Y | Y | Y | Y | Y | N | Y | Un | Y | Y | Y |
| Choi 1996 [14] | N | Y | N | Y | Y | Y | Y | Y | N | N | Y | Y | Y | Y |
| Czub 2014 [15] | N | N | N | Y | Y | Y | Y | Y | N | N | Y | Y | Y | Y |
| Denno 2005 [16] | N | Y | N | Y | Y | Y | Y | Y | Y | Un | Un | Y | Y | Y |
| DuBois 1988 [17] | N | Y | N | Y | Y | Y | Y | Y | Y | Y | Un | Y | Y | Y |
| Duman 2015 [18] | Y | Y | N | Y | Y | Y | Y | Y | N | Un | Un | Y | Y | Y |
| Fan 1993 [19] | N | Y | N | Y | N | Y | Y | Y | N | Y | Un | Y | Y | Y |
| Hossain 1991 [20] | Un | Y | N | Y | Y | Y | Y | Y | Y | Y | Un | Y | Y | Y |
| Huicho 1993 [21] | Y | Y | N | Y | Y | Y | Y | Y | N | Un | Un | Y | Y | Y |
| Huicho 1997 [22] | Y | Y | N | Y | Y | Y | Y | Y | Y | Un | Un | Y | Y | Y |
| Ismail 1994 [23] | Y | Y | N | Y | Y | Y | Y | Y | Y | Y | Un | Y | Y | Y |
| Jindal 1991 [24] | Un | N | N | Y | Y | Y | Y | Y | N | Y | Un | Y | Y | Y |
| Khan 2006 [25] | Y | Y | N | Y | Y | Y | Y | Y | Y | Y | Un | Y | Y | Y |
| Kim 2022 [26] | Un | N | Y | Y | Y | Y | Y | N | Y | Un | Un | Y | Y | Y |
| Koplan 1980 [27] | N | Y | N | Un | N | Y | Y | N | Y | Y | Un | Y | Y | Y |
| Korzeniowski 1979 [28] | Y | Y | N | Y | Y | Y | Y | Y | Y | Un | Un | Y | Y | Y |
| Lai 2016 [29] | N | Y | Y | Y | Y | Y | Y | Y | N | Y | N | Y | Y | Y |
| Lever 2021 [30] | N | Y | N | Un | N | Un | Y | N | N | Y | Un | Y | Y | Y |
| Loosli 1985 [31] | N | Y | N | Y | Y | Y | Y | Y | Y | Un | Un | Y | Y | Y |
| McIver 2001 [32] | N | N | N | Y | Y | Y | Y | Y | Y | Un | Un | N | Y | N |
| McNeely 1996 [33] | N | Y | N | Y | Y | Y | Y | Y | Y | Un | Un | Y | Y | Y |
| Mercado 2011 [34] | Y | N | N | Y | Y | Y | Y | Y | Y | Un | Un | Y | Y | Y |
| Miller 1994 [35] | N | N | Y | Un | Y | Y | Y | Y | Y | N | Y | N | Y | Y |
| Mshana 2010 [36] | Un | N | N | Y | Y | Y | Y | N | Y | Y | Un | Y | Y | Y |
| Nordlander 1990 [37] | Y | Y | N | Y | Y | Y | Y | N | Y | Y | Un | Y | Y | Y |
| Paccagnini 1987 [38] | N | N | Un | Y | Y | Y | Y | Y | N | Un | Un | Y | Y | Y |
| Park 2019 [39] | N | Y | Y | Y | Y | Y | Y | Y | Y | Un | Un | Y | Y | Y |
| Patwari 1993 [40] | Y | Y | N | Y | Y | Y | Y | Y | Y | Y | Un | Y | Y | Y |
| Pender 2022 [41] | N | Y | N | Y | Y | Y | Y | N | Y | Y | Un | Y | Y | Y |
| Scerpella 1994 [42] | N | N | N | Y | Y | Y | Y | Y | Y | Y | Y | Y | Y | Y |
| Sebodo 1978 [43] | N | Y | N | Y | Y | Y | Y | Y | N | Y | Un | Y | Y | Y |
| Shastri 2008 [44] | N | Y | N | Y | Y | Y | Y | Y | N | Un | Un | Y | Y | Y |
| Siegel 1987 [45] | N | Y | N | Y | Y | Y | Y | Y | Y | Un | Un | Y | Y | Y |
| Sykora 2010 [46] | N | Y | N | Un | Y | Y | Y | Y | Y | Y | Y | Y | Y | Y |
| Tribble 2008 [47] | N | N | N | Y | Y | Y | Y | Y | Y | Y | Y | Y | Y | Y |
| Venkataraman 2003 [48] | N | N | N | Y | Y | Y | Y | Y | N | Un | Un | Y | Y | Y |
| Wang 2014 [49] | N | Y | Y | Y | Y | Y | Y | N | Y | Y | Un | Y | Y | Y |

Y = Yes; N = No; Un = Unclear

^1^ Study populations were considered representative if they included children with diarrhea presenting to health services in low-resource settings (exclusions: inpatient services only, bloody diarrhea/dysentery only, adults only, international travelers, military personnel, volunteers, high-income setting).

^2^ Selection criteria given in methods.

^3^ Molecular methods (i.e. PCR) for identification were considered accurate reference standards; bacterial culture was considered insensitive.

^4^ Index (biomarker) and referent (culture/molecular) test(s) performed on the same sample, or two samples (e.g. whole stool and rectal swabs) were specified to have been collected at the same time.

^5^ Everyone included received reference test.

^6^ Reference test received was not conditional on result of index test.

^7^ Index test did not form part of the reference test.

^8^ Execution of the index test(s) was described in sufficient detail in the methods to permit replication of the test.

^9^ Execution of the reference standard test was described in sufficient detail in the methods to permit replication of the test.

^10^ Index (biomarker) test interpreted without knowledge of reference (culture/molecular) result (assumed true for index tests by microscopy since they were necessarily completed before the reference test).

^11^ Reference result interpreted without knowledge of index test.

^12^ Clinical data was comparable to normal practices during interpretation (i.e. basic details of participant characteristics and diarrheal illness).

^13^ Uninterpretable/intermediate tests were not possible or were reported.

^14^ All patients received both index and reference tests and/or reasons for withdrawals were provided.

**References**

1. Ahn JS, Seo SI, Kim J, et al. Efficacy of stool multiplex polymerase chain reaction assay in adult patients with acute infectious diarrhea. World J Clin Cases **2020**; 8:3708–3717.

2. Alvarado T. Faecal leucocytes in patients with infectious diarrhoea. Transactions of the Royal Society of Tropical Medicine and Hygiene **1983**; 77:316–320.

3. Aly SM, El-Zawawy LA, Said DE, Fathy FM, Mohamed ON. The utility of lactoferrin in differentiating parasitic from bacterial infections. J Egypt Soc Parasitol **2005**; 35:1149–1162.

4. Alzaher MZ, Almugahwi AA, Almulla AA, Almeer HH, Alshammasi MM, El-Badry AA. Diagnostic yield of stool culture and probable predictive factors, A single-center experience. Acta Biomed **2022**; 93:e2022302.

5. Ascher DP, Ednsada-Corpus R. Clinical and Laboratory Predictors of Bacterial Diarrhea in a Tropical Environment. Military Medicine **1991**; 156:74–76.

6. Ashraf H, Beltinger J, Alam NH, et al. Evaluation of Faecal Occult Blood Test and Lactoferrin Latex Agglutination Test in Screening Hospitalized Patients for Diagnosing Inflammatory and Non-Inflammatory Diarrhoea in Dhaka, Bangladesh. Gastroenterologia **2008**; 76:256–261.

7. Bardhan PK, Beltinger J, Beltinger RW, Hossain A, Mahalanabis D, Gyr K. Screening of patients with acute infectious diarrhoea: evaluation of clinical features, faecal microscopy, and faecal occult blood testing. Scand J Gastroenterol **2000**; 35:54–60.

8. Beltinger J, Walther R, Bardhan P, Mahalanabis D, Gyr K. Immunological Testing for Occult Blood in Patients with Acute Infectious Diarrhea (Can It Improve the Specificity of the Guaiac Test?). Dig Dis Sci **1997**; 42:366–371.

9. Berger C, Loitsch SM, Hartmann F, Stein J. 657 Comparative Evaluation of Fecal Calprotectin and S100A12 as Non-Invasive Markers in Predicting Microbiological Diagnosis for Acute Bacterial Diarrhea: Prospective Multicenter Study. Gastroenterology **2010**; 5 Supplement 1:S-88.

10. Bodhidatta L, Vjthayasai N, Eimpokalarp B, Pitarangsi C, Serichantalergs O, Isenbarger D. Bacterial enteric pathogens in children with acute dysentery in Thailand: increasing importance of quinolone-resistant Campylobacter. The Southeast Asian journal of tropical medicine and public health **2002**; 33:752–7.

11. Bouckenooghe, Steffen R, Bouckenooghe AR, et al. Markers of enteric inflammation in enteroaggregative Escherichia coli diarrhea in travelers. The American Journal of Tropical Medicine and Hygiene **2000**; 62:711–713.

12. Caprioli A, Pezzella C, Morelli R, et al. Enteropathogens associated with childhood diarrhea in Italy. The Pediatric Infectious Disease Journal **1996**; 15:876.

13. Chang H, Zhang L, Ge Y, et al. A Hospital-based Case-control Study of Diarrhea in Children in Shanghai. The Pediatric Infectious Disease Journal **2017**; 36:1057–1063.

14. Choi SW, Park CH, Silva TM, Zaenker EI, Guerrant RL. To culture or not to culture: fecal lactoferrin screening for inflammatory bacterial diarrhea. Journal of Clinical Microbiology **1996**; 34:928–932.

15. Czub E, Nowak JK, Moczko J, et al. Comparison of fecal pyruvate kinase isoform M2 and calprotectin in acute diarrhea in hospitalized children. Sci Rep **2014**; 4:4769.

16. Denno DM, Stapp JR, Boster DR, et al. Etiology of Diarrhea in Pediatric Outpatient Settings. The Pediatric Infectious Disease Journal **2005**; 24:142.

17. DuBois D, Binder L, Nelson B. Usefulness of the stool Wight’s stain in the emergency department. The Journal of Emergency Medicine **1988**; 6:483–486.

18. Duman M, Gencpinar P, Biçmen M, et al. Fecal calprotectin: can be used to distinguish between bacterial and viral gastroenteritis in children? The American Journal of Emergency Medicine **2015**; 33:1436–1439.

19. Fan K, Morris AJ, Reller LB. Application of rejection criteria for stool cultures for bacterial enteric pathogens. Journal of Clinical Microbiology **1993**; 31:2233–2235.

20. Hossain MA, Albert MJ. Effect of duration of diarrhoea and predictive values of stool leucocytes and red blood cells in the isolation of different serogroups or serotypes of Shigella. Transactions of the Royal Society of Tropical Medicine and Hygiene **1991**; 85:664–666.

21. Huicho L, Sanchez D, Contreras M, et al. Occult blood and fecal leukocytes as screening tests in childhood infectious diarrhea: an old problem revisited. The Pediatric Infectious Disease Journal **1993**; 12:474.

22. Huicho L, Garaycochea V, Uchima N, Zerpa R, Guerrant RL. Fecal lactoferrin, fecal leukocytes and occult blood in the diagnostic approach to childhood invasive diarrhea. Pediatr Infect Dis J **1997**; 16:644–647.

23. Ismail. Indicators for antibiotic therapy in invasive bacterial diarrhoea - Record details - Embase. 1994. Available at: https://www.embase.com/records?subaction=viewrecord&rid=2&page=1&id=L25048116. Accessed 10 July 2023.

24. Jindal N, Arora S. Role of faecal leucocytes in the diagnostic evaluation of acute diarrhoea. Indian J Med Sci **1991**; 45:261–264.

25. Khan AI, Huq S, Malek MA, et al. Analysis of fecal leukocytes and erythrocytes in Shigella infections in urban Bangladesh. Southeast Asian J Trop Med Public Health **2006**; 37:747–754.

26. Kim HJ. Efficacy of Fecal Calprotectin Combined With Stool Hemoglobin in Differentiating Bacterial Origin in Acute Gastroenteritis. Pediatric Emergency Care **2022**; 38:e670.

27. Koplan JeffreyP, Jane Benfari Ferraro M, Fineberg HarveyV, Rosenberg MarkL. VALUE OF STOOL CULTURES. The Lancet **1980**; 316:413–416.

28. Korzeniowski OM, Barada FA, Rouse JD, Guerrant RL. Value of examination for fecal leukocytes in the early diagnosis of shigellosis. Am J Trop Med Hyg **1979**; 28:1031–1035.

29. Lai C-C, Ji D-D, Wu F-T, et al. Etiology and Risk Factors of Acute Gastroenteritis in a Taipei Emergency Department: Clinical Features for Bacterial Gastroenteritis. Journal of Epidemiology **2016**; 26:216–223.

30. Lever RA, Tapper L, Skarbek S, Chiodini PL, Armstrong M, Bailey RL. Predictors of aetiology and outcomes of acute gastrointestinal illness in returning travellers: a retrospective cohort analysis. BMC Infectious Diseases **2021**; 21:599.

31. Loosli J, Gyr K, Stalder H, et al. Etiology of Acute Infectious Diarrhea in a Highly Industrialized Area of Switzerland. Gastroenterology **1985**; 88:75–79.

32. J. McIverw GH Peter White, Jennifer C Doultree, Michael Catton, William D Rawlinson, Christopher. Diagnosis of Enteric Pathogens in Children with Gastroenteritis. Pathology **2001**; 33:353–358.

33. McNeely WS, Dupont HL, Mathewson JJ, Oberhelman RA, Ericsson CD. Occult blood versus fecal leukocytes in the diagnosis of bacterial diarrhea: a study of U.S. travelers to Mexico and Mexican children. Am J Trop Med Hyg **1996**; 55:430–433.

34. Mercado EH, Ochoa TJ, Ecker L, et al. Fecal Leukocytes in Children Infected with Diarrheagenic Escherichia coli. Journal of Clinical Microbiology **2011**; 49:1376–1381.

35. Miller JR, Barrett LJ, Kotloff K, Guerrant RL. A Rapid Test for Infectious and Inflammatory Enteritis. Archives of Internal Medicine **1994**; 154:2660–2664.

36. Mshana SE, Joloba ML, Kakooza A, Kaddu-Mulindwa D. Role of microscopic examination of stool specimens in the diagnosis of campylobacter infection from children with acute diarrhoea in Kampala, Uganda. Tanzania Journal of Health Research **2010**; 12:100–103.

37. Nordlander E, Phuphaisan S, Bodhidatta L, Arthur J, Echeverria P. Microscopic examination of stools and a latex slide agglutination test for the rapid identification of bacterial enteric infections in khmer children. Diagnostic Microbiology and Infectious Disease **1990**; 13:273–276.

38. Paccagnini S, Fontana M, Ceriani R, et al. Occult blood and faecal leucocyte tests in acute infectious diarrhoea in children. Lancet **1987**; 1:442.

39. Park Y, Son M, Jekarl DW, Choi HY, Kim SY, Lee S. Clinical Significance of Inflammatory Biomarkers in Acute Pediatric Diarrhea. Pediatr Gastroenterol Hepatol Nutr **2019**; 22:369–376.

40. Patwari AK, Deb M, Dudeja M, Jayasheela M, Agarwal A, Singh P. Clinical and laboratory predictors of invasive diarrhoea in children less than five years old. J Diarrhoeal Dis Res **1993**; 11:211–216.

41. Pender MA, Smith T, Brintz BJ, et al. Weather variables as important clinical predictors of bacterial diarrhoea among international travellers. Journal of Travel Medicine **2022**; 29:taac012.

42. Scerpella EG, Okhuysen PC, Mathewson JJ, et al. Evaluation of a New Latex Agglutination Test for Fecal Lactoferrin in Travelers’ Diarrhea. Journal of Travel Medicine **1994**; 1:68–71.

43. Sebodo T, Soetarjo, Sadjimin T, Soenarto Y, Sanborn WR. Study on the etiology of diarrhea. Journal of Tropical Pediatrics and Environmental Child Health **1978**; 24:107–109.

44. Shastri YM, Bergis D, Povse N, et al. Prospective Multicenter Study Evaluating Fecal Calprotectin in Adult Acute Bacterial Diarrhea. The American Journal of Medicine **2008**; 121:1099–1106.

45. Siegel D, Cohen P, Neighbor M, et al. Predictive value of stool examination in acute diarrhea. Archives of pathology & laboratory medicine **1987**; 111:715–8.

46. Sýkora J, Siala K, Huml M, Varvařovská J, Schwarz J, Pomahačová R. Evaluation of faecal calprotectin as a valuable non-invasive marker in distinguishing gut pathogens in young children with acute gastroenteritis. Acta Paediatrica **2010**; 99:1389–1395.

47. Tribble DR, Baqar S, Pang LW, et al. Diagnostic Approach to Acute Diarrheal Illness in a Military Population on Training Exercises in Thailand, a Region of Campylobacter Hyperendemicity. Journal of Clinical Microbiology **2008**; 46:1418–1425.

48. Venkataraman S, Ramakrishna BS, Kang G, Rajan DP, Mathan VI. Faecal lactoferrin as a predictor of positive faecal culture in south Indian children with acute diarrhoea. Annals of Tropical Paediatrics **2003**; 23:9–13.

49. Wang Y, Zhang TP, Xiao HL, Qi HY, Yin CH. Formulation of an early warning infectivity score system for adult patients with acute bacterial diarrhea. Biomed Environ Sci **2014**; 27:65–69.
